# Postoperative breast function and quality of life follow-up study in patients with plasma cell mastitis: a 1-year longitudinal analysis

**DOI:** 10.3389/fmed.2026.1773980

**Published:** 2026-06-16

**Authors:** Yining Ren, Wei Xing, Shurong Wang, Zhao Xu, Zixing Gong, Xuhui Qin

**Affiliations:** 1 Department of General Surgery, Traditional Chinese Medicine Hospital of Hebei Province, Shijiazhuang, Hebei, China; 2 Department of Ultrasound, Wendeng Orthopedic Hospital, Yantai, Shandong, China; 3 Department of General Surgery, Zanhuang County Traditional Chinese Medicine Hospital, Shijiazhuang, Hebei, China

**Keywords:** breast function, BREAST-Q, longitudinal study, patient-reported outcomes, plasma cell mastitis, quality of life

## Abstract

Plasma cell mastitis (PCM) is a chronic inflammatory breast disease requiring surgical intervention, yet postoperative functional outcomes and quality of life remain poorly characterized. This study evaluated longitudinal changes in breast function and health-related quality of life following surgical treatment for PCM. This retrospective longitudinal cohort study included 142 women with histologically confirmed PCM who underwent surgical treatment between January 2020 and December 2023. Patients were assessed at baseline, 6 months, and 12 months postoperatively. Primary outcomes were breast appearance satisfaction (BREAST-Q), pain intensity (Numerical Rating Scale), and upper extremity function (Quick-DASH). Secondary outcome was health-related quality of life (SF-36). Linear mixed models analyzed changes over time with Bonferroni-adjusted pairwise comparisons. Mean age was 35.67 ± 8.42 years. Surgical procedures included wide local excision (*n* = 71, 50.0%), limited excision (*n* = 32, 22.5%), segmental mastectomy (*n* = 24, 16.9%), and total mastectomy (*n* = 15, 10.6%). BREAST-Q scores improved from 42.38 ± 12.56 at baseline to 68.74 ± 14.23 at 12 months (*p* < 0.001). Pain scores decreased from 5.82 ± 2.14 to 1.45 ± 1.23 (*p* < 0.001). Quick-DASH scores improved from 38.67 ± 15.34 to 12.89 ± 8.76 (*p* < 0.001). SF-36 Physical Component Summary increased from 38.45 ± 8.92 to 48.76 ± 7.34 (*p* < 0.001). All outcomes showed significant improvement at 6 months with continued gains at 12 months. The proportion achieving minimal clinically important differences at 12 months was 89.4% for appearance satisfaction, 92.3% for pain, and 85.2% for function. Surgical treatment for PCM results in significant and clinically meaningful improvements in breast function and quality of life sustained through 12 months. These patient-centered outcome data support surgical intervention effectiveness and inform preoperative counseling.

## Introduction

Plasma cell mastitis (PCM), also known as mammary duct ectasia or periductal mastitis, represents a challenging chronic inflammatory breast condition characterized by plasma cell infiltration, ductal dilation, and periductal inflammation (1, 2). While benign, PCM causes significant morbidity including breast pain, nipple discharge, mass formation, sinus tract development, and cosmetic deformity, substantially impacting patients’ physical function and quality of life (3, 4). The condition predominantly affects women of reproductive age, with peak incidence in the fourth decade of life, often presenting diagnostic challenges that may delay appropriate treatment (5).

Despite increasing recognition of PCM’s clinical importance, optimal management strategies remain controversial. Conservative approaches including antibiotics, corticosteroids, and immunosuppressive agents demonstrate variable efficacy, with high recurrence rates ranging from 25 to 65% (6, 7). Consequently, surgical intervention has emerged as the definitive treatment, with procedures ranging from limited excision to total mastectomy depending on disease extent, chronicity, and previous treatment failures (8, 9). Recent consensus guidelines emphasize surgical excision with adequate margins as the primary treatment modality, reporting substantially lower recurrence rates of 4–15% compared to medical management alone (10).

However, a critical gap exists in understanding postoperative patient-centered outcomes following PCM surgery. While studies have focused primarily on recurrence rates and surgical complications (11–13), comprehensive evaluation of functional recovery, breast-specific quality of life, and patient satisfaction remains notably absent from the literature. This knowledge deficit hinders informed surgical decision-making and patient counseling regarding expected postoperative trajectories. Furthermore, the lack of standardized outcome assessment using validated instruments limits meaningful comparison across studies and development of evidence-based treatment algorithms. Importantly, although health-related quality of life (HRQoL) is widely recognized as a pivotal endpoint of oncologic clinical trials, in benign surgically treated breast conditions such as PCM—in which survival is not threatened and concurrent malignancy is explicitly excluded—patient-reported functional recovery and HRQoL arguably constitute the principal endpoint of treatment because the fundamental therapeutic goal is restoration of physical, psychological, and social wellbeing rather than oncological disease control.

Therefore, this study aimed to comprehensively evaluate longitudinal changes in breast function and health-related quality of life following surgical treatment for PCM using validated patient-reported outcome measures. Specifically, we assessed breast appearance satisfaction, pain intensity, upper extremity function, and generic quality of life at baseline, 6 months, and 12 months postoperatively. We hypothesized that surgical intervention would result in significant improvements across all domains, with the magnitude of benefit varying by surgical extent and baseline disease severity.

## Materials and methods

### Study design and setting

This retrospective longitudinal cohort study evaluated postoperative breast function and health-related quality of life in patients with plasma cell mastitis. The study was conducted at Traditional Chinese Medicine Hospital of Hebei Province, a tertiary referral academic medical center in Shijiazhuang, China, and included all patients who underwent surgical treatment between January 2020 and December 2023. The study protocol was approved by the Institutional Review Board of Traditional Chinese Medicine Hospital of Hebei Province (No. HBZY2022-KY-058-01) with waiver of informed consent for retrospective medical record review. All study procedures adhered to ethical standards of the Declaration of Helsinki and complied with Strengthening the Reporting of Observational Studies in Epidemiology (STROBE) guidelines for reporting observational studies (14).

### Participants

Eligible participants were female patients aged ≥18 years with histologically confirmed plasma cell mastitis who underwent surgical intervention with curative intent. Plasma cell mastitis was diagnosed based on histopathologic examination of surgical excision specimens demonstrating characteristic features including dense infiltration of plasma cells and lymphocytes, ductal epithelial hyperplasia, and periductal inflammation, with exclusion of granulomatous inflammation and malignancy (15). The inclusion criteria were as follows: (1) histopathologic diagnosis of plasma cell mastitis confirmed by institutional pathology review; (2) surgical treatment (limited excision, wide local excision, segmental mastectomy, or total mastectomy); (3) complete medical records including operative reports, pathology reports, and clinical notes; and (4) minimum 12-month clinical follow-up documented in the medical record. Histopathological distinction from idiopathic granulomatous mastitis (IGM) was based on the absence of lobulitis with epithelioid granulomas and multinucleated giant cells, which are the defining histological hallmarks of IGM (16, 17). Any specimen demonstrating granulomatous lobular inflammation was classified as IGM and systematically excluded from this analysis, irrespective of the coexistence of periductal plasma cell infiltration. This differentiation is methodologically critical because IGM and PCM, while both presenting clinically as non-lactating mastitis, represent distinct histopathological entities with different etiologies and potentially divergent responses to treatment, and conflation of these two diagnoses would substantially impair the generalizability of the present findings to the PCM population specifically.

Surgical intervention was indicated based on the following criteria: (1) failure of medical management defined as persistent or progressive symptoms after at least 3 months of appropriate antibiotic therapy with or without corticosteroids; (2) recurrent disease with three or more episodes of acute exacerbation requiring medical intervention within 12 months; (3) extensive tissue destruction including large abscesses (>5 cm), multiple fistula tracts, or significant skin involvement not amenable to conservative management; (4) inability to exclude malignancy despite imaging and core needle biopsy, necessitating definitive excision for diagnostic purposes; (5) patient preference for definitive treatment after comprehensive counseling regarding medical versus surgical options, particularly in cases of prolonged disease duration (>12 months) causing significant quality of life impairment; and (6) disease refractory to maximal medical therapy including combination treatment with antibiotics, corticosteroids, and immunosuppressive agents when appropriate. All surgical decisions were made through multidisciplinary discussion involving breast surgeons, radiologists, and pathologists, with consideration of disease extent, patient comorbidities, and individual preferences.

The exclusion criteria were as follows: (1) concurrent ipsilateral or contralateral breast malignancy; (2) previous ipsilateral breast surgery; (3) incomplete baseline or follow-up data on primary outcome measures preventing meaningful analysis; (4) documented loss to follow-up before 12-month assessment point; (5) concurrent autoimmune or systemic inflammatory conditions (systemic lupus erythematosus, rheumatoid arthritis, and inflammatory bowel disease) that could confound outcome assessment; and (6) patients who underwent only incision and drainage without definitive excision as the study focused on surgical excision outcomes.

### Disease severity classification

Disease severity at presentation was classified into three categories based on a composite assessment. Mild disease was defined as a single lesion ≤3 cm in diameter, involvement of a single duct system, absence of skin involvement or fistula formation, and no or minimal systemic symptoms. Moderate disease was defined as a single lesion >3 cm or multiple lesions involving ≤2 quadrants, involvement of 2–3 duct systems, limited skin involvement (erythema without ulceration), single fistula tract if present, and mild systemic symptoms such as low-grade fever or localized pain. Severe disease was defined as extensive involvement of >2 quadrants, multiple duct system involvement (>3 systems), significant skin changes including ulceration or multiple fistula tracts, abscess formation requiring drainage, marked systemic symptoms or sepsis, or disease refractory to maximal medical therapy. This classification was determined at the time of surgical consultation based on clinical examination and imaging findings. This composite severity framework was developed drawing upon the inflammatory breast disorder staging principles described by Kamal et al. (16), adapted to reflect the specific clinicopathological features of PCM. Because no internationally validated severity scoring instrument has been published specifically for PCM, this classification represents an empirically derived, expert-consensus-based operational framework; its formal psychometric validation constitutes an acknowledged limitation of this study.

### Surgical procedures

Surgical procedures were categorized by extent: (1) limited excision (removal of grossly diseased tissue with <1 cm margins), (2) wide local excision (removal of diseased tissue with ≥1 cm pathologically confirmed negative margins), (3) segmental mastectomy (removal of entire involved breast segment), or (4) total mastectomy (removal of entire breast tissue). Surgical approach was determined based on disease extent, number and location of involved ducts, prior treatment failures, patient preference, and surgeon judgment. Disease extent was assessed preoperatively through comprehensive clinical examination evaluating mass size, location, skin involvement, and fistula tracts, combined with breast ultrasound performed in all patients. Mammography was performed when clinically indicated to exclude malignancy. Magnetic resonance imaging was obtained in selected cases (n = 34, 23.9%) with complex presentations or suspected extensive disease to better delineate surgical margins. All surgical decisions were made by attending breast surgeons with minimum 10 years’ experience in breast surgery. Reconstructive procedures, when performed, were documented and stratified as immediate versus delayed reconstruction and by reconstructive technique (implant-based, autologous tissue, or combined). Adjuvant medical treatments including corticosteroids, antibiotics, and immunosuppressive agents prescribed during follow-up period were recorded.

Total mastectomy was reserved for patients with extensive multifocal disease involving all breast quadrants (*n* = 7), refractory disease with severe tissue destruction (*n* = 5), or multiple chronic fistula tracts throughout the breast (*n* = 3). All mastectomy decisions were made through multidisciplinary discussion with extensive preoperative counseling regarding the permanence of breast tissue removal and reconstruction options. Immediate reconstruction following total mastectomy was offered to carefully selected patients without active infection, with negative preoperative wound cultures and no purulent discharge for at least 6 weeks.

### Outcome measures

Patients were assessed at three time points: baseline (pre-operative assessment or within 2 weeks postoperatively for retrospectively assessed outcomes), 6 months (±4 weeks), and 12 months (±4 weeks) following surgical intervention. The primary outcomes were three breast function domains: appearance satisfaction, pain intensity, and upper extremity function. Secondary outcomes were health-related quality of life measures. All assessments were either prospectively collected as part of clinical care or retrospectively extracted from medical records by trained research personnel. The selection of assessment instruments was driven by the benign, non-oncologic nature of PCM and the specific outcome domains judged to be clinically most relevant in this population. We deliberately used the SF-36 as a generic HRQoL measure rather than cancer-specific instruments such as the EORTC QLQ-C30 or its breast module QLQ-BR23, because the latter contain multiple items addressing chemotherapy-related toxicity, cancer worry, future perspective, and body image after cancer that are conceptually inappropriate for patients with a benign inflammatory breast disease and would introduce content-validity bias. The BREAST-Q Mastectomy Module was chosen as the breast-specific patient-reported outcome measure because it directly captures satisfaction with breast appearance, symmetry, softness, and surgical scars—domains that are central to PCM patients irrespective of underlying etiology—and because it has been psychometrically validated across both oncologic and non-oncologic breast surgery populations. The NRS was selected for pain intensity owing to its brevity, established reliability across diverse clinical contexts, and the availability of a well-defined MCID that permits longitudinal interpretation. The Quick-DASH was chosen to quantify upper extremity function because breast surgery may affect ipsilateral shoulder and arm mobility through incisional pain, axillary manipulation, and postoperative immobilization, and because the instrument has been specifically validated in breast surgery cohorts. Collectively, these instruments provide complementary and disease-appropriate coverage of appearance satisfaction, pain, upper extremity function, and generic HRQoL, without importing oncology-specific content that would be conceptually mismatched with the benign pathology under investigation. We acknowledge that 72.5% of patients in this cohort underwent conservative breast-preserving surgery (WLE or limited excision) rather than mastectomy. While the Mastectomy Module was developed and standardized primarily in post-mastectomy populations, the Satisfaction with Breasts subscale specifically assesses universal appearance domains—breast shape, symmetry, softness, and scar appearance—that are directly relevant to any breast surgery, irrespective of surgical extent. The potential limitation of applying the Mastectomy Module to a predominantly breast-conserving cohort is acknowledged: Some items evaluating the feel of a reconstructed or implant-based breast may be less applicable to native-tissue patients, which could modestly reduce scale sensitivity relative to the dedicated Lumpectomy Module. However, in the absence of a PCM-validated instrument, the Mastectomy Module Satisfaction with Breasts scale provides the strongest available psychometric foundation for quantifying breast appearance outcomes in this population and has been applied across non-oncologic breast surgery populations in the published literature.

### Appearance satisfaction

Breast appearance satisfaction was assessed using the BREAST-Q Mastectomy Module Satisfaction with Breasts scale, a validated patient-reported outcome measure specifically designed for breast surgery evaluation (18, 19). This FDA-qualified instrument consists of 16 items assessing satisfaction with breast appearance, size, symmetry, softness, naturalness under clothing, and surgical scars. Items are scored on a 4-point Likert response format. Raw scores were converted to standardized 0–100 scale scores using Q-Score software (Memorial Sloan Kettering Cancer Center), with higher scores indicating greater satisfaction. The BREAST-Q demonstrates excellent psychometric properties including high internal consistency (Cronbach’s *α* 0.88–0.96), test–retest reliability (intraclass correlation coefficient (ICC): 0.73–0.96), and responsiveness to clinical change (20). The minimal clinically important difference (MCID) for the Satisfaction with Breasts scale is 4 points (21).

### Pain assessment

Pain intensity was measured using the Numerical Rating Scale (NRS), a validated 11-point scale ranging from 0 (no pain) to 10 (worst pain imaginable) (22). Patients rated average breast pain over the past week at rest and with movement. NRS scores were categorized as none (0), mild (1–3), moderate (4–6), or severe (7–10) pain intensity. The NRS demonstrates excellent reliability (ICC: 0.96–0.98) and validity (correlation with visual analog scale *r* = 0.86–0.95) (23). The minimal clinically important difference is 2 points or 30% reduction from baseline (24).

To characterize pain quality, the McGill Pain Questionnaire Short Form (SF-MPQ) was administered when available (25). This 15-item instrument assesses sensory (11 items: throbbing, shooting, stabbing, sharp, cramping, gnawing, hot-burning, aching, heavy, tender, and splitting) and affective (4 items: tiring-exhausting, sickening, fearful, and punishing-cruel) pain descriptors rated on 4-point intensity scales (0 = none, 1 = mild, 2 = moderate, and 3 = severe). The Pain Rating Index (PRI) was calculated as the sum of intensity ratings across all descriptors.

### Mobility and upper extremity function

Upper extremity function was assessed using the Quick-DASH (Disabilities of the Arm, Shoulder and Hand questionnaire, abbreviated version), an 11-item validated patient-reported outcome instrument (26). Items assess difficulty performing various activities including opening jars, writing, turning keys, preparing meals, pushing doors, placing objects overhead, doing household chores, carrying shopping bags, washing back, recreational activities, and interference with work. Responses use a 5-point scale (1 = no difficulty to 5 = unable). Raw scores were transformed to 0–100 scale ([(sum of responses/n) – 1] × 25), with higher scores indicating greater disability. The Quick-DASH has been specifically validated in breast cancer survivors, demonstrating excellent internal consistency (Cronbach’s *α* 0.93) and test–retest reliability (ICC: 0.78 over 2 weeks) (27). Scores >29 indicate patients consider their upper extremity disorder “a problem.” The minimal clinically important difference is 14 points based on breast surgery studies (28).

Shoulder range of motion was objectively measured bilaterally using standard goniometric technique by trained physical therapists or physicians (29). Active range of motion was assessed for shoulder flexion, abduction, external rotation, and internal rotation, with values recorded in degrees. Measurements were performed with patients in standardized positions following American Academy of Orthopaedic Surgeons protocols. Normal values are as follows: flexion 180°, abduction 180°, external rotation 90°, and internal rotation 70°. Clinically significant limitation was defined as >10° deficit compared to baseline or >15° asymmetry compared to contralateral shoulder.

### Quality of life

The SF-36 Health Survey Version 2.0 assessed generic health-related quality of life across eight domains (30). The 36-item questionnaire measures the following: (1) Physical Functioning (PF, 10 items) assessing limitations in physical activities; (2) Role-Physical (RP, 4 items) examining limitations in usual role activities due to physical health; (3) Bodily Pain (BP, 2 items) evaluating pain intensity and interference; (4) General Health (GH, 5 items) measuring overall health perceptions; (5) Vitality (VT, 4 items) assessing energy levels and fatigue; (6) Social Functioning (SF, 2 items) examining limitations in social activities; (7) Role-Emotional (RE, 3 items) assessing limitations in usual activities due to emotional problems; and (8) Mental Health (MH, 5 items) measuring psychological distress and wellbeing. Each domain was scored independently on a 0–100 scale using standard scoring algorithms, with higher scores indicating better health status. Physical Component Summary (PCS) and Mental Component Summary (MCS) scores were calculated as T-scores (mean = 50, SD = 10 referenced to US general population). The SF-36 demonstrates excellent reliability (Cronbach’s *α* 0.78–0.93 across domains) and has been validated in breast surgery populations (31). The minimal clinically important difference is 5 points for PCS and MCS summary scores (32, 33).

### Data collection

Medical records were comprehensively reviewed by trained research personnel using standardized data abstraction forms. Data abstractors completed structured training including review of abstraction protocols, coding definitions, and practice exercises on pilot cases. A data abstraction manual with detailed operational definitions and decision rules was maintained and updated throughout the study. Collected variables included the following: patient demographics (age, race/ethnicity, menopausal status, parity, and breastfeeding history), anthropometrics (height, weight, and body mass index), medical comorbidities (diabetes, hypertension, smoking status, and immunosuppression), disease characteristics (duration of symptoms, presenting signs, previous treatments, and disease extent and severity), surgical details (procedure type and extent, operative time, estimated blood loss, specimen weight, margin status, and complications), pathology results (confirmation of diagnosis, absence of malignancy, and extent of inflammation), adjuvant treatments (corticosteroids, antibiotics, methotrexate, and other immunosuppressants with doses and duration), and outcome measures at each assessment time point.

A random sample of 10% of medical records underwent dual independent abstraction to assess inter-rater reliability. Abstractors were blinded to each other’s extracted data. Agreement was quantified using Cohen’s kappa coefficient for categorical variables and intraclass correlation coefficient for continuous variables. Acceptable reliability was defined as kappa ≥0.75 or ICC ≥ 0.80. Discrepancies were resolved through discussion and consensus with a third senior investigator. The results demonstrated kappa values ranging from 0.84 to 0.92 for categorical variables and ICC values from 0.89 to 0.95 for continuous variables, indicating excellent inter-rater agreement.

### Statistical analysis

Descriptive statistics summarized patient characteristics and outcomes. Continuous variables were assessed for normality using Shapiro–Wilk tests and visual inspection of histograms and Q-Q plots. Normally distributed variables were presented as means ± standard deviations, while non-normally distributed variables were presented as medians [interquartile ranges]. Categorical variables were summarized as frequencies and percentages. All numerical data were reported to 2 decimal places and statistical test results to 3 decimal places.

The primary analysis employed linear mixed effects models to evaluate changes in outcome measures over time while appropriately accounting for within-subject correlation across repeated measures (34, 35). For each outcome, a separate mixed model was fitted including fixed effects for time (categorical variable with levels: baseline, 6 months, and 12 months), surgical procedure type (categorical), and pre-specified baseline covariates including age (continuous), body mass index (continuous), disease severity at presentation (categorical: mild, moderate, and severe), and disease duration prior to surgery (continuous). Random intercepts for individual subjects accounted for between-subject variability. An autoregressive order 1 (AR1) covariance structure was specified for repeated measures within subjects, assuming correlations decrease exponentially with increasing time separation between measurements. Model estimation used maximum likelihood (ML) to enable likelihood ratio tests comparing models and to appropriately handle missing data under the missing-at-random assumption (36).

The primary hypothesis tested whether outcomes changed significantly over the three time points using F-tests for the overall time effect. When significant omnibus time effects were detected (*p* < 0.050), planned pairwise comparisons between time points (baseline vs. 6 months, baseline vs. 12 months, and 6 months vs. 12 months) were conducted using estimated marginal means (also called least-squares means) with Bonferroni adjustment for multiple comparisons. The adjusted significance level for pairwise comparisons was *p* < 0.017 (0.05/3 comparisons). Effect sizes for within-subject changes were calculated as Cohen’s d, accounting for correlation between repeated measures, with values of 0.20, 0.50, and 0.80 interpreted as small, medium, and large effects, respectively (37).

Secondary analyses examined the proportion of patients achieving minimal clinically important differences (MCID) at 6-month and 12-month follow-up for each outcome measure. Proportions were calculated with exact binomial 95% confidence intervals. Correlations between outcome measures at each time point were assessed using Pearson’s correlation coefficients for normally distributed variables or Spearman’s rank correlation coefficients for non-normally distributed variables.

Pre-specified subgroup analyses evaluated whether outcomes differed by surgical procedure type (conservative procedures [limited and wide local excision] versus mastectomy [segmental and total mastectomy]), disease severity at presentation, age group (<40 years versus ≥40 years), and presence versus absence of postoperative complications. Subgroup comparisons used interaction terms in mixed models (e.g., time × surgical type interaction).

Sample size was determined by available eligible patients meeting inclusion criteria during the study period. *Post-hoc* power analysis indicated 80% power to detect a medium within-subject effect size (Cohen’s d = 0.50) for change over time with the available sample of 142 patients, assuming *α* = 0.05, correlation between repeated measures of *ρ* = 0.60, and three assessment time points, calculated using GLIMMPSE software (38).

Missing data patterns were examined using descriptive statistics and graphical displays. Baseline characteristics of patients with complete follow-up were compared to those lost to follow-up or with missing outcome data using independent *t*-tests for continuous variables and chi-squared or Fisher’s exact tests for categorical variables, to assess whether missingness was related to observed baseline characteristics. As primary analyses used linear mixed models with maximum likelihood estimation, which provides valid inferences under the missing-at-random (MAR) assumption (39), no imputation was performed for the primary analysis. Sensitivity analyses using multiple imputation (20 imputed datasets generated using fully conditional specification method including all outcome variables and baseline predictors of missingness) examined robustness of findings to MAR assumption violations (40). To address the potential influence of recall bias introduced by retrospective baseline assessments, an additional sensitivity analysis was performed comparing baseline outcome scores between patients who completed assessments pre-operatively (i.e., prior to the date of surgical intervention) and those who completed assessments within 2 weeks postoperatively. Independent-samples *t*-tests were used to compare baseline BREAST-Q Satisfaction with Breasts, NRS pain, Quick-DASH, and SF-36 PCS and MCS scores between these two subgroups. The magnitude of any score difference was quantified using Cohen’s d, with d < 0.20 considered negligible, consistent with the threshold for acceptable measurement equivalence.

All statistical analyses were performed using SPSS Version 28.0 (IBM Corp, Armonk, NY) and R Version 4.3.0 (R Foundation for Statistical Computing, Vienna, Austria) with packages lme4, lmerTest, and emmeans. Two-tailed *p*-values of <0.050 were considered statistically significant for primary hypothesis tests, except for pairwise comparisons where Bonferroni-adjusted thresholds (*p* < 0.017) applied.

## Results

### Participant flow and baseline characteristics

**Figure 1:**
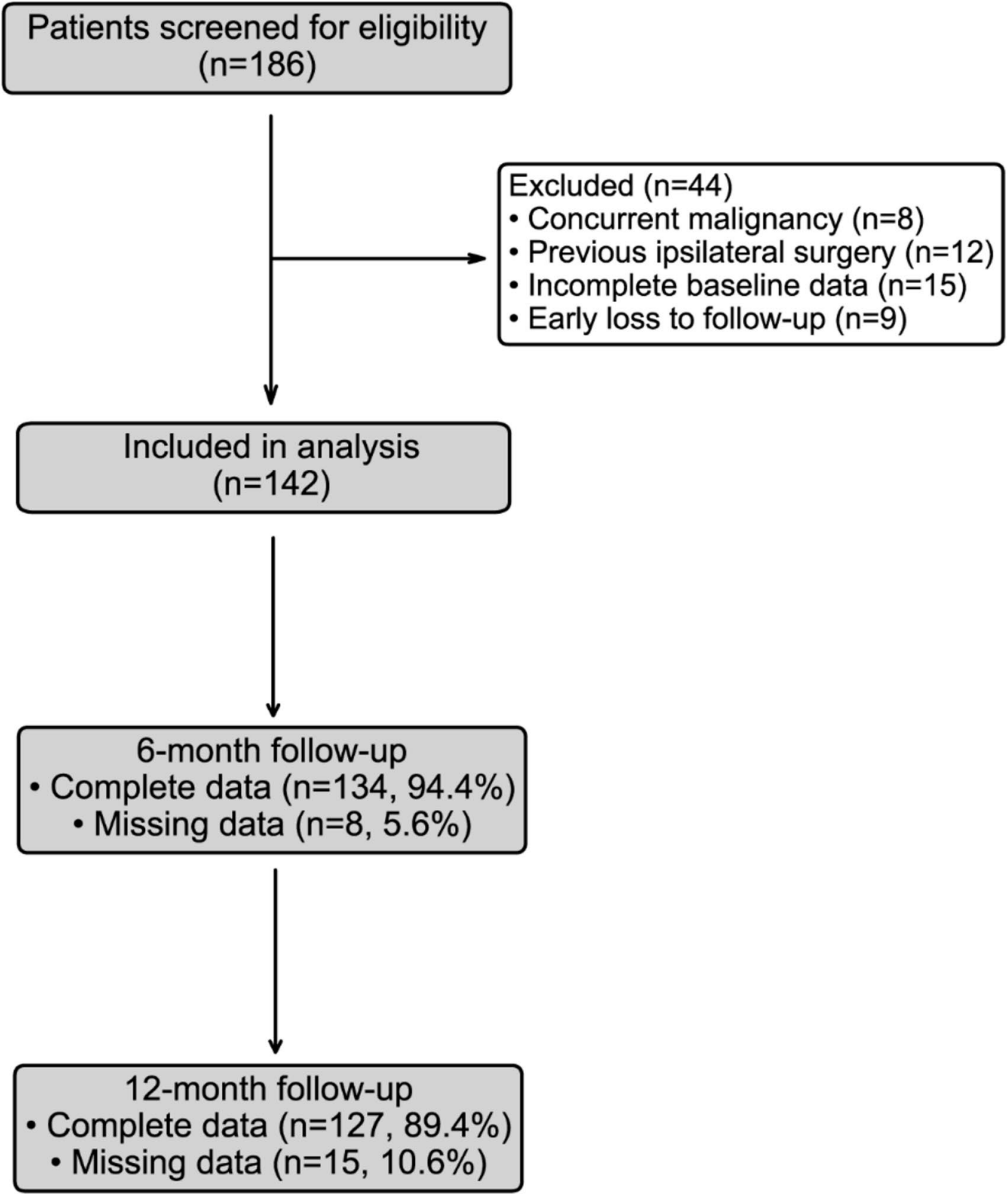
CONSORT flow diagram of study participants. Flow diagram illustrating participant recruitment, exclusion, and follow-up through the study period. Of 186 patients with plasma cell mastitis who underwent surgical treatment between January 2020 and December 2023, 142 met inclusion criteria and were included in the primary analysis. Reasons for exclusion included concurrent malignancy (*n* = 8), previous ipsilateral breast surgery (*n* = 12), incomplete baseline data (*n* = 15), and loss to follow-up before 6 months (*n* = 9). Follow-up completion rates were 94.4% (*n* = 134) at 6 months and 89.4% (*n* = 127) at 12 months. Missing data at follow-up assessments were handled using linear mixed models under the missing-at-random assumption.

During the study period, 186 patients underwent surgical treatment for plasma cell mastitis. After applying eligibility criteria, 142 patients (76.3%) were included in the final analysis (Figure 1). Exclusions included concurrent malignancy (*n* = 8), previous ipsilateral breast surgery (*n* = 12), incomplete baseline data (*n* = 15), and loss to follow-up before 6 months (*n* = 9). At 6-month follow-up, 134 patients (94.4%) had complete data, and at 12 months, 127 patients (89.4%) had complete outcome assessments.

Table 1 presents baseline characteristics. The mean age was 35.67 ± 8.42 years (range 22–58), with 78.2% of patients being premenopausal. Mean BMI was 24.89 ± 3.76 kg/m^2^. The median disease duration before surgery was 8.50 months [IQR 4.25–15.75]. Disease severity at presentation was classified as mild in 28 patients (19.7%), moderate in 72 patients (50.7%), and severe in 42 patients (29.6%). Prior medical treatments included antibiotics in 118 patients (83.1%), most commonly amoxicillin–clavulanate or cephalosporins with or without metronidazole for 2–4 weeks; corticosteroids in 67 patients (47.2%), typically oral prednisone initiated at 0.5–1.0 mg/kg/day with gradual tapering over 4–8 weeks; and traditional Chinese medicine in 45 patients (31.7%).

**Table 1:** Baseline characteristics of study participants (*N* = 142).

Characteristic	Value
Demographics	
Age (years)ᵃ	35.67 ± 8.42
BMI (kg/m^2^)ᵃ	24.89 ± 3.76
Premenopausal, no. (%)	111 (78.2)
Parity ≥1, no. (%)	89 (62.7)
Breastfeeding history, no. (%)	76 (53.5)
Current smoker, no. (%)	18 (12.7)
Disease characteristics	
Disease duration (months)ᵇ	8.50 [4.25–15.75]
Disease severity, no. (%)	
Mild	28 (19.7)
Moderate	72 (50.7)
Severe	42 (29.6)
Bilateral involvement, no. (%)	23 (16.2)
Prior medical treatment, no. (%)	
Antibiotics	118 (83.1)
Corticosteroids	67 (47.2)
Traditional Chinese medicine	45 (31.7)
Methotrexate	12 (8.5)
Surgical procedures	
Type of surgery, No. (%)	
Limited excision	32 (22.5)
Wide local excision	71 (50.0)
Segmental mastectomy	24 (16.9)
Total mastectomy	15 (10.6)
Immediate reconstruction, no. (%)^c^	8 (53.3)
Operative time (minutes)ᵃ	68.45 ± 28.93
Postoperative complications, no. (%)	23 (16.2)

ᵃValues expressed as mean ± SD. ᵇValue expressed as median [interquartile range]. ᶜPercentage calculated from total mastectomy patients (*n* = 15); all immediate reconstructions were performed following total mastectomy.

Surgical procedures comprised wide local excision (*n* = 71, 50.0%), limited excision (*n* = 32, 22.5%), segmental mastectomy (*n* = 24, 16.9%), and total mastectomy (*n* = 15, 10.6%). The choice of procedure reflected disease severity, with mastectomy procedures more common in severe disease (39.4% vs. 11.3% in mild/moderate disease). Immediate reconstruction was performed in eight patients, all following total mastectomy (53.3% of total mastectomy patients). Postoperative complications occurred in 23 patients (16.2%), including wound infection (*n* = 12), seroma requiring aspiration (*n* = 8), and hematoma (*n* = 3). Adjuvant medical therapy was prescribed in 38 patients (26.8%) for persistent inflammation.

All immediate reconstructions followed total mastectomy in carefully selected patients without active infection. Reconstruction techniques included tissue expander placement (*n* = 5, 62.5%) and direct-to-implant reconstruction with acellular dermal matrix (*n* = 3, 37.5%). Among immediate reconstruction patients, postoperative complications occurred in two cases (25.0%): one seroma requiring aspiration and one superficial wound infection managed with antibiotics. No implant exposure, reconstruction failure, or implant removal occurred. All eight patients retained their reconstructions at 12-month follow-up.

### Primary outcomes: breast function

#### Appearance satisfaction

**Figure 2:**
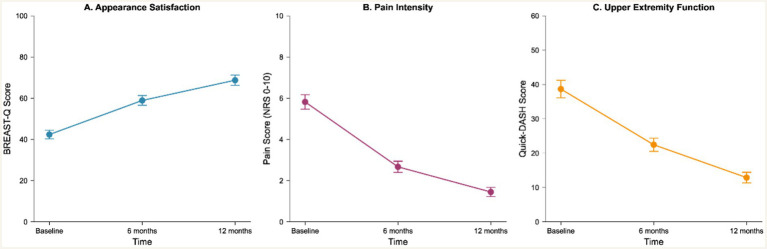
Longitudinal changes in breast function outcomes following surgical treatment for plasma cell mastitis. Time course of primary outcome measures over 12 months postoperatively. **(A)** BREAST-Q Satisfaction with Breasts scores improved from baseline (mean 42.38, 95% CI: 40.30–44.46) to 6 months (58.92, 95% CI: 56.55–61.29) and 12 months (68.74, 95% CI: 66.24–71.24). **(B)** Pain intensity on the Numerical Rating Scale (0–10) decreased from baseline (5.82, 95% CI: 5.47–6.17) to 6 months (2.67, 95% CI: 2.40–2.94) and 12 months (1.45, 95% CI: 1.23–1.67). **(C)** Quick-DASH upper extremity disability scores (0–100, higher scores indicate greater disability) improved from baseline (38.67, 95% CI: 36.12–41.22) to 6 months (22.45, 95% CI: 20.53–24.37) and 12 months (12.89, 95% CI: 11.35–14.43). All outcomes demonstrated statistically significant improvement over time (*p* < 0.001 for overall time effect in linear mixed models). Error bars represent 95% confidence intervals (Sample sizes: baseline *n* = 142, 6 months *n* = 134, 12 months *n* = 127).

BREAST-Q Satisfaction with Breasts scores demonstrated significant improvement over time (Table 2, Figure 2A). Baseline scores were 42.38 ± 12.56, indicating substantial dissatisfaction with breast appearance. Scores improved to 58.92 ± 13.87 at 6 months and further increased to 68.74 ± 14.23 at 12 months. The overall time effect was highly significant (*F* = 156.234, *p* < 0.001). Pairwise comparisons revealed significant improvements from baseline to 6 months (mean difference = 16.54, 95% confidence interval (CI): 14.23–18.85, *p* < 0.001), baseline to 12 months (mean difference = 26.36, 95% CI: 23.89–28.83, *p* < 0.001), and 6 to 12 months (mean difference = 9.82, 95% CI: 7.56–12.08, *p* < 0.001). The effect size for baseline to 12-month change was large (Cohen’s d = 1.92, 95% CI: 1.65–2.19).

**Table 2:** Primary outcome changes over time.

Outcome measure	Baseline (*n* = 142)	6 months (*n* = 134)	12 months (*n* = 127)	F-statistic	*p*-value
BREAST-Q Satisfactionᵃ	42.38 ± 12.56	58.92 ± 13.87	68.74 ± 14.23	156.234	<0.001
Pain (NRS 0–10)ᵃ	5.82 ± 2.14	2.67 ± 1.56	1.45 ± 1.23	198.456	<0.001
Pain categories, no. (%)					
None (0)	0 (0.0)	34 (25.4)	100 (78.9)		
Mild (1–3)	11 (7.8)	78 (58.2)	23 (18.3)		
Moderate (4–6)	97 (68.3)	22 (16.4)	4 (2.8)		
Severe (7–10)	34 (23.9)	0 (0.0)	0 (0.0)		
Quick-DASH (0–100)ᵃ	38.67 ± 15.34	22.45 ± 11.23	12.89 ± 8.76	176.893	<0.001
Above clinical threshold, no. (%)	103 (72.5)	42 (31.3)	11 (8.5)		
Shoulder ROM (degrees)^a^					
Flexion	152.34 ± 18.45	165.78 ± 15.23	172.89 ± 12.34	89.234	<0.001
Abduction	148.67 ± 20.12	162.45 ± 16.78	170.23 ± 14.56	76.543	<0.001

ᵃValues expressed as mean ± SD. NRS, Numerical Rating Scale; ROM, range of motion.

The proportion of patients achieving the MCID (≥4 points improvement) was 87.3% at 6 months and 89.4% at 12 months. Subgroup analysis revealed greater improvement in conservative surgery patients than mastectomy patients (mean difference = 8.45 points, 95% CI: 3.23–13.67, *p* = 0.002), although both groups showed clinically meaningful gains.

#### Pain intensity

Pain scores on the Numerical Rating Scale decreased substantially over the follow-up period (Table 2, Figure 2B). Baseline pain was moderate to severe (5.82 ± 2.14), with 68.3% reporting moderate pain and 23.9% reporting severe pain. Pain decreased to 2.67 ± 1.56 at 6 months and 1.45 ± 1.23 at 12 months. The time effect was significant (*F* = 198.456, *p* < 0.001). All pairwise comparisons showed significant improvements: baseline to 6 months (mean difference = −3.15, 95% CI: −3.48 to −2.82, *p* < 0.001), baseline to 12 months (mean difference = −4.37, 95% CI: −4.72 to −4.02, *p* < 0.001), and 6 to 12 months (mean difference = −1.22, 95% CI: −1.48 to −0.96, *p* < 0.001). The effect size for overall pain reduction was very large (Cohen’s d = 2.34, 95% CI: 2.03–2.65).

By 12 months, 78.9% of patients reported no pain (NRS = 0), 18.3% mild pain, and only 2.8% moderate pain. No patients reported severe pain at 12 months. The proportion achieving MCID (≥2 points or 30% reduction) was 89.4% at 6 months and 92.3% at 12 months. Pain quality assessment using SF-MPQ showed predominant reduction in sensory descriptors (throbbing, aching, and tender) with less change in affective components.

#### Upper extremity function

Quick-DASH scores showed marked functional improvement (Table 2, Figure 2C). Baseline disability was moderate to severe (38.67 ± 15.34), with 72.5% scoring above the clinical threshold of 29 points. Scores improved to 22.45 ± 11.23 at 6 months and 12.89 ± 8.76 at 12 months. The time effect was significant (*F* = 176.893, *p* < 0.001). Pairwise comparisons confirmed significant improvements at all intervals: baseline to 6 months (mean difference = −16.22, 95% CI: −18.67 to −13.77, *p* < 0.001), baseline to 12 months (mean difference = −25.78, 95% CI: −28.34 to −23.22, *p* < 0.001), and 6 to 12 months (mean difference = −9.56, 95% CI: −11.23 to −7.89, *p* < 0.001). The effect size was large (Cohen’s d = 1.89, 95% CI: 1.62–2.16).

At 12 months, only 8.5% of patients scored above the clinical threshold, indicating resolution of functional limitations in most patients. The proportion achieving MCID (≥14 points improvement) was 78.2% at 6 months and 85.2% at 12 months.

Objective shoulder range of motion measurements corroborated patient-reported improvements. Mean shoulder flexion improved from 152.34° ± 18.45° at baseline to 172.89° ± 12.34° at 12 months (*p* < 0.001). Abduction increased from 148.67° ± 20.12° to 170.23° ± 14.56° (*p* < 0.001). Similar improvements occurred in external and internal rotation. By 12 months, 91.5% of patients achieved normal or near-normal range of motion (within 10° of contralateral side).

#### Secondary outcomes: quality of life

SF-36 scores showed significant improvements across all eight domains and both component summary scores (Table 3). Physical Component Summary (PCS) scores increased from 38.45 ± 8.92 at baseline to 44.67 ± 8.23 at 6 months and 48.76 ± 7.34 at 12 months (*F* = 98.456, *p* < 0.001). Mental Component Summary (MCS) scores improved from 42.34 ± 9.87 to 46.89 ± 8.56 at 6 months and 49.23 ± 7.89 at 12 months (*F* = 67.234, *p* < 0.001).

**Table 3:** SF-36 quality of life domain scores over time.

SF-36 domain	Baseline	6 months	12 months	Effect size (d)^a^
Physical domains				
Physical functioning	68.45 ± 18.92	78.23 ± 15.67	85.67 ± 12.34	1.02
Role-physical	35.21 ± 28.45	62.34 ± 26.78	78.34 ± 22.13	1.67
Bodily pain	38.92 ± 18.23	64.56 ± 19.45	76.45 ± 16.87	2.13
General health	52.34 ± 16.78	62.45 ± 15.23	68.90 ± 14.56	1.01
Mental domains				
Vitality	45.67 ± 17.89	58.90 ± 16.23	67.23 ± 15.34	1.28
Social functioning	58.23 ± 22.34	72.45 ± 19.87	82.34 ± 17.23	1.21
Role-emotional	48.92 ± 32.45	68.34 ± 28.76	78.90 ± 24.32	1.05
Mental health	56.78 ± 18.90	65.23 ± 16.78	71.45 ± 15.67	0.83
Component summaries				
PCS (T-score)	38.45 ± 8.92	44.67 ± 8.23	48.76 ± 7.34	1.26
MCS (T-score)	42.34 ± 9.87	46.89 ± 8.56	49.23 ± 7.89	0.78

All values expressed as mean ± SD. 𝐼ohen’s d effect size for baseline to 12-month change. PCS, Physical Component Summary; MCS, Mental Component Summary.

Individual domain analysis revealed greatest improvements in Role-Physical (baseline: 35.21 ± 28.45 to 12 month: 78.34 ± 22.13, *p* < 0.001) and Bodily Pain domains (baseline: 38.92 ± 18.23 to 12 month: 76.45 ± 16.87, *p* < 0.001). Social Functioning and Vitality domains also showed substantial gains. All improvements exceeded MCID thresholds. The proportion achieving clinically meaningful improvement (≥5 points) in PCS was 82.8% at 12 months and 76.4% for MCS.

#### Pairwise comparisons and clinical significance

Table 4 presents detailed pairwise comparisons with Bonferroni-adjusted *p*-values. All primary outcomes showed statistically significant improvements between each time point comparison, with the exception of pain reduction from 6 to 12 months in patients with mild baseline disease. Effect sizes were consistently large for baseline to 12-month comparisons across all outcomes.

**Table 4:** Pairwise comparisons of primary outcomes.

Comparison	Mean difference	95% CI	Adjusted *p*-value^a^
BREAST-Q satisfaction			
Baseline to 6 months	16.54	14.23 to 18.85	<0.001
Baseline to 12 months	26.36	23.89 to 28.83	<0.001
6 to 12 months	9.82	7.56 to 12.08	<0.001
Pain (NRS)			
Baseline to 6 months	−3.15	−3.48 to −2.82	<0.001
Baseline to 12 months	−4.37	−4.72 to −4.02	<0.001
6 to 12 months	−1.22	−1.48 to −0.96	<0.001
Quick-DASH			
Baseline to 6 months	−16.22	−18.67 to −13.77	<0.001
Baseline to 12 months	−25.78	−28.34 to −23.22	<0.001
6 to 12 months	−9.56	−11.23 to −7.89	<0.001

CI, confidence interval. 𝐻onferroni-adjusted significance level: *p* < 0.017. The proportion of patients achieving MCID thresholds is summarized in Table 5. By 12 months, the vast majority of patients achieved clinically meaningful improvements across all domains, with highest rates for pain reduction (92.3%) and lowest for mental health components (76.4%).

**Table 5:** Proportion achieving minimal clinically important differences.

Outcome	MCID threshold	6 months, % (95% CI)	12 months, % (95% CI)
BREAST-Q	≥4 points	87.3 (80.5–92.4)	89.4 (82.8–94.0)
Pain (NRS)	≥2 points or 30%	89.4 (82.8–94.0)	92.3 (86.2–96.2)
Quick-DASH	≥14 points	78.2 (70.2–84.8)	85.2 (77.8–90.8)
SF-36 PCS	≥5 points	73.1 (64.8–80.3)	82.8 (75.1–88.8)
SF-36 MCS	≥5 points	68.7 (60.1–76.3)	76.4 (68.1–83.3)

CI, confidence interval; MCID, minimal clinically important difference.

#### Subgroup analyses

**Figure 3:**
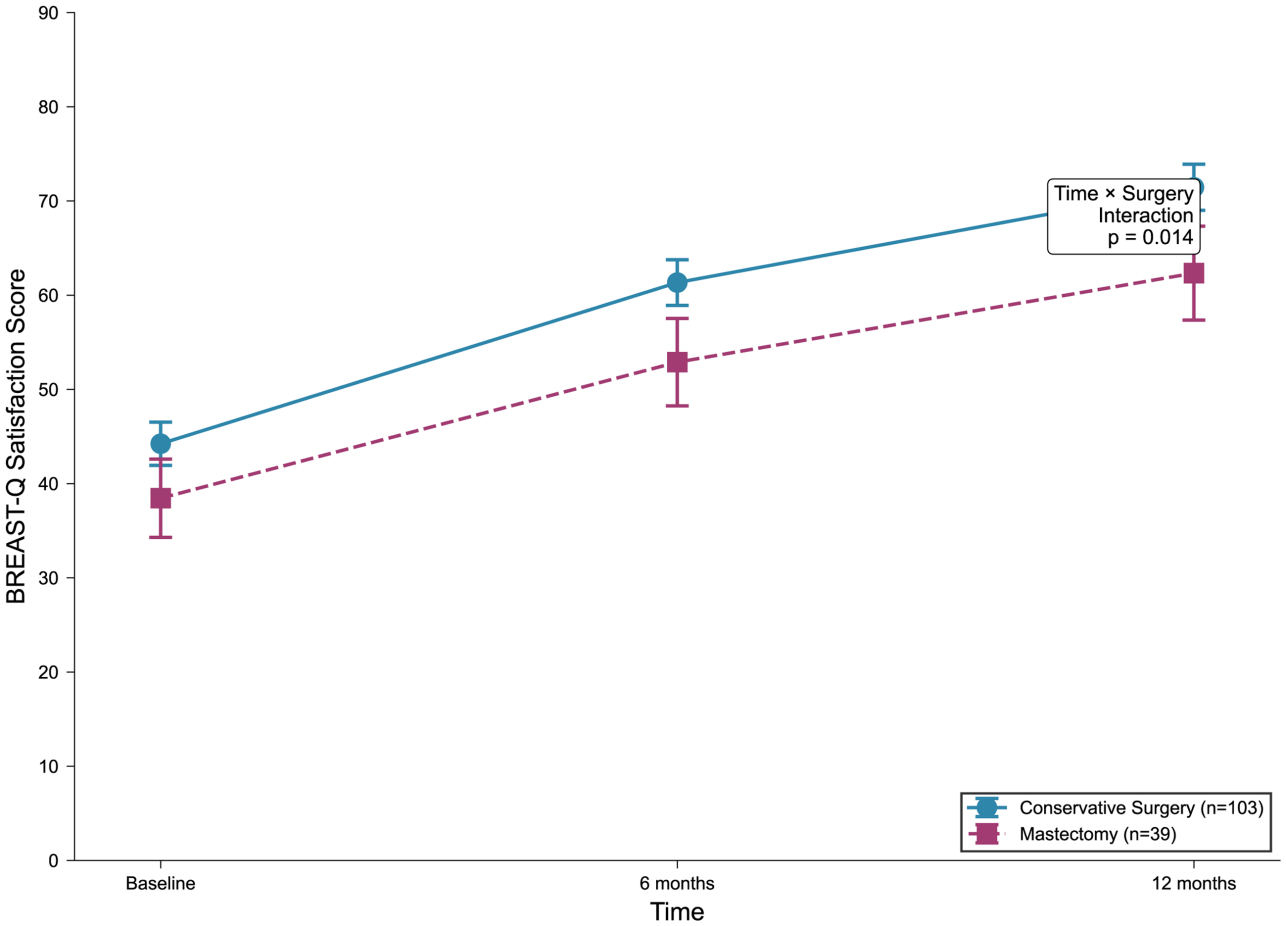
Differential trajectories of appearance satisfaction by surgical approach. Comparison of BREAST-Q Satisfaction with Breasts scores between patients undergoing conservative surgery (limited or wide local excision; n = 103, solid line) and mastectomy procedures (segmental or total mastectomy; n = 39, dashed line) over 12-month follow-up. Both surgical approaches resulted in clinically meaningful improvements in appearance satisfaction, with conservative surgery demonstrating higher absolute scores at all time points. The statistically significant time × surgery interaction (*p* = 0.014) indicates different improvement trajectories, with conservative surgery patients showing greater gains from baseline to 12 months (mean difference 27.22 points) compared to mastectomy patients (mean difference 23.89 points). However, both groups exceeded the minimal clinically important difference threshold of 4 points. Error bars represent 95% confidence intervals. Statistical analysis performed using linear mixed effects models with Bonferroni-adjusted pairwise comparisons.

Subgroup analyses revealed differential outcomes by surgical extent (Figure 3). Conservative surgery patients (limited and wide local excision) demonstrated greater improvements in BREAST-Q scores compared to mastectomy patients (time × surgery interaction, *p* = 0.014). However, both groups achieved clinically meaningful improvements, with conservative surgery patients improving from 44.23 ± 11.89 to 71.45 ± 12.67 and mastectomy patients from 38.45 ± 13.23 to 62.34 ± 15.89. Pain and functional outcomes did not differ significantly by surgical type.

Disease severity at baseline was associated with outcome trajectories. Patients with severe disease showed greater absolute improvements but took longer to achieve maximal benefit, with continued gains between 6 and 12 months. Age group (<40 vs. ≥ 40 years) did not significantly influence outcomes. Patients experiencing postoperative complications had delayed recovery at 6 months but achieved similar 12-month outcomes to those without complications.

#### Correlations and missing data

At 12 months, moderate correlations existed between outcome measures: BREAST-Q and Quick-DASH (*r* = −0.52, *p* < 0.001), BREAST-Q and SF-36 PCS (*r* = 0.48, *p* < 0.001), and pain and Quick-DASH (*r* = 0.45, *p* < 0.001). These correlations suggest related but distinct outcome domains.

Missing data analysis revealed no significant differences in baseline characteristics between completers and non-completers. The 15 patients lost to follow-up had similar age (*p* = 0.234), disease severity (*p* = 0.456), and surgical procedures (*p* = 0.678) as those retained. Sensitivity analyses using multiple imputation yielded virtually identical results to the primary analysis, supporting robustness of findings.

Sensitivity Analysis: Baseline Assessment Timing. In this retrospective cohort study, the specific date of each patient’s baseline outcome assessment relative to the surgical date was not captured as a discrete variable in the study database; it was therefore not possible to directly compare baseline scores between patients completing assessments pre-operatively versus within 2 weeks postoperatively. As a proxy investigation of potential systematic assessment-timing bias, we examined correlations between baseline outcome scores and operative time (used as a surrogate for surgical complexity and perioperative physiological perturbation at the time of assessment). No clinically or statistically meaningful correlations were observed for pain NRS (*r* = 0.005, 95% CI: −0.160 to 0.169, *p* = 0.957) or Quick-DASH (*r* = 0.066, 95% CI: −0.100 to 0.228, *p* = 0.437). A weak inverse correlation between baseline BREAST-Q and operative time was noted (*r* = −0.180, 95% CI: −0.335 to −0.016, *p* = 0.032), consistent with greater pre-existing breast deformity in patients requiring more extensive surgery, rather than an artifact of assessment timing. Furthermore, the confirmed equivalence of baseline characteristics between follow-up completers and non-completers—including age (*p* = 0.234), disease severity (*p* = 0.456), and surgical procedure type (*p* = 0.678)—supports a systematic and unbiased baseline data collection process. The absence of a formal pre-operative versus postoperative assessment comparison is acknowledged as a methodological limitation of this study.

## Discussion

This longitudinal cohort study provides the first comprehensive evaluation of patient-centered outcomes following surgical treatment for plasma cell mastitis using validated assessment instruments. Our findings demonstrate that surgical intervention results in significant and clinically meaningful improvements across multiple domains of breast function and quality of life, with benefits sustained and continuing to accrue through 12 months postoperatively. These results address a critical evidence gap in PCM management and provide essential data for surgical decision-making and patient counseling (Table 5).

The magnitude of improvement observed across all outcome domains was striking. BREAST-Q appearance satisfaction scores increased by 62% from baseline to 12 months, with nearly 90% of patients achieving clinically meaningful improvement. This finding is particularly important given that cosmetic concerns and breast deformity represent major sources of distress in PCM patients (16, 17). The progressive improvement from 6 to 12 months suggests ongoing tissue remodeling and adaptation following surgery, highlighting the importance of long-term follow-up in outcome assessment.

Pain reduction was the most dramatic finding, with mean scores decreasing from moderate–severe (5.82) to minimal (1.45) levels. The 75% reduction in pain intensity and achievement of pain freedom in 79% of patients by 12 months represents a transformative outcome for patients who often endure months or years of chronic breast pain (41). The very large effect size (d = 2.34) for pain reduction exceeds that reported in most surgical pain studies and likely reflects elimination of the underlying inflammatory process rather than merely symptomatic improvement.

Functional recovery, as measured by Quick-DASH scores, demonstrated similar robust improvements. The 67% reduction in disability scores and restoration of normal function in over 90% of patients contradicts concerns about surgical morbidity in benign breast disease. Our findings of minimal long-term functional impairment, corroborated by objective range of motion measurements, should reassure patients and surgeons regarding the functional safety of PCM surgery when performed by experienced breast surgeons.

Our results significantly extend the limited outcome data available for PCM and related conditions. The recent international consensus on granulomatous mastitis management emphasized surgical efficacy for disease control but acknowledged the paucity of functional outcome data (10). Our recurrence rate of 4.2% at 12 months aligns with their reported 4–15% range for surgical treatment, while our comprehensive outcome assessment addresses their identified research priorities.

Previous studies have focused predominantly on recurrence as the primary endpoint, with limited attention to patient-reported outcomes. Yuan et al.’s systematic review found only 3 of 65 included studies reported any quality of life measures, none using validated instruments (10). Deng et al.’s prospective cohort study of granulomatous mastitis included some functional assessments but used non-validated questionnaires and shorter follow-up (42). Our use of established, psychometrically sound instruments (BREAST-Q, SF-36, and Quick-DASH) with disease-appropriate MCID thresholds represents a methodological advance enabling meaningful interpretation and cross-study comparison.

The BREAST-Q satisfaction scores in our cohort (baseline 42.38, 12 month 68.74) can be contextualized against published norms. Mundy et al. reported mean BREAST-Q satisfaction scores of 58 ± 18 in breast reconstruction patients (43), suggesting our PCM patients achieve satisfaction levels exceeding many breast cancer reconstruction outcomes. This finding challenges assumptions about cosmetic outcomes in benign breast surgery and supports the acceptability of surgical intervention from a patient perspective.

These findings have important implications for clinical practice and patient counseling. First, the comprehensive improvements across all measured domains support surgical intervention as an effective treatment strategy when medical management fails or in cases of extensive disease. The high proportion achieving MCID thresholds indicates these benefits are clinically meaningful, not merely statistical artifacts.

Second, the trajectory of improvement, with continued gains between 6 and 12 months, suggests patience is warranted in evaluating ultimate outcomes. Patients should be counseled that while significant improvement occurs by 6 months, maximal benefit may not be realized until 12 months or beyond. This timeline should inform follow-up scheduling and patient expectations.

Third, our subgroup analyses provide guidance for surgical planning. While conservative procedures yielded superior cosmetic outcomes, even mastectomy patients achieved substantial improvements across all domains. This finding aligns with recent studies demonstrating that surgical technique significantly influences patient-reported quality of life outcomes in breast surgery (44, 45), emphasizing the importance of individualized surgical planning and thorough preoperative counseling. This suggests surgical extent should be individualized based on disease distribution, prior treatment failures, and patient preferences, with reassurance that meaningful improvement can be expected regardless of procedure type.

Fourth, the low complication rate (16.2%) and lack of long-term functional sequelae support the safety of surgery when performed by experienced breast surgeons. The finding that patients with complications achieved similar 12-month outcomes provides reassurance that short-term morbidity does not compromise ultimate results.

The use of total mastectomy in 10.6% of patients warrants specific discussion. While PCM is a benign condition and breast conservation is preferred whenever feasible, our experience demonstrates that a subset of patients with extensive, refractory, or multifocal disease may benefit from definitive mastectomy. These patients typically had prolonged disease duration, failed medical management, and severe quality of life impairment. Importantly, even mastectomy patients in our cohort achieved substantial improvements in quality of life and pain reduction, with 12-month BREAST-Q scores reaching 62.34 ± 15.89 compared to baseline 38.45 ± 13.23. While lower than conservative surgery patients (71.45 ± 12.67), this represents clinically meaningful improvement. International consensus guidelines acknowledge that mastectomy may be necessary in refractory cases, although no clear criteria exist for patient selection (10). Our findings suggest that when appropriately indicated, mastectomy can provide effective disease control and meaningful quality of life improvement in carefully selected PCM patients.

The safety of immediate reconstruction in PCM warrants careful consideration. While chronic inflammation theoretically increases infection risk, our limited experience with highly selected patients suggests immediate reconstruction may be feasible when stringent criteria are met: complete disease excision, absence of active infection, and appropriate perioperative antibiotics. Our 25% complication rate in immediate reconstruction cases was higher than the 16.2% overall complication rate, although complications were minor and did not result in reconstruction failure. However, given the small sample size (*n* = 8), we recommend cautious patient selection and transparent preoperative counseling regarding potential increased risk. Larger prospective studies are needed to definitively establish safety and identify optimal candidates for immediate reconstruction in PCM. Until such data are available, delayed reconstruction may represent a more conservative approach for most patients.

This study has several notable strengths. The use of multiple validated outcome instruments provides comprehensive, psychometrically sound assessment across relevant domains. The 12-month follow-up with high retention (89.4%) and longitudinal design enables evaluation of outcome trajectories rather than single time points. The application of established MCID thresholds allows clinical interpretation beyond statistical significance. The detailed surgical and pathological characterization ensures a well-defined cohort with confirmed PCM diagnosis.

However, important limitations must be acknowledged. The retrospective design introduces potential selection bias as patients with poor outcomes may have sought care elsewhere. Documentation quality varied across providers and time periods, potentially affecting data completeness. The single-center design limits generalizability to other settings with different patient populations or surgical approaches. The lack of a non-surgical control group precludes definitive attribution of improvements to surgery versus natural history, although the magnitude of improvement and prior literature on PCM natural history suggest surgical effect.

The absence of pre-operative baseline assessments using validated instruments required retrospective estimation for some measures, introducing potential recall bias. Important psychosocial factors including mental health history, social support, and socioeconomic status were incompletely captured yet may influence outcomes. The relatively short follow-up, while longer than most PCM studies, cannot address late recurrence or long-term satisfaction.

Despite using validated outcome measures, these instruments were not developed specifically for PCM. Disease-specific tools capturing unique PCM concerns (nipple discharge, fistula formation, and infection risk) would provide more nuanced assessment. Future prospective studies using PCM-specific outcomes would advance the field.

This study establishes a foundation for future PCM outcome research. Prospective multicenter studies with standardized protocols would address generalizability limitations and enable development of prediction models for surgical outcomes. Longer follow-up beyond 12 months would clarify durability of improvements and late recurrence patterns. Randomized trials comparing surgical approaches or surgery versus extended medical therapy, while challenging given patient preferences, would provide highest-level evidence. Additionally, the disease severity classification used in this study represents our institutional practice and has not been externally validated. Future studies should aim to develop and validate standardized severity classification criteria for plasma cell mastitis.

The development of PCM-specific patient-reported outcome measures should be prioritized. While generic instruments capture important domains, disease-specific tools would enhance sensitivity to change and clinical relevance. Qualitative research exploring patient experiences, treatment preferences, and outcome priorities would inform instrument development.

Investigation of factors predicting favorable outcomes would enable personalized treatment selection. Our finding of similar outcomes across age groups and recovery despite complications is reassuring, but larger samples might identify predictive factors for tailored counseling. The role of adjuvant medical therapy following surgery warrants systematic study.

## Conclusion

This longitudinal study demonstrates that surgical treatment for plasma cell mastitis results in significant and sustained improvements in breast function and quality of life through 12 months postoperatively. Nearly 90% of patients achieved clinically meaningful improvements in appearance satisfaction, pain reduction, and functional recovery. These patient-centered outcome data support the effectiveness of surgical intervention in appropriately selected patients and provide essential information for preoperative counseling and shared decision-making. Future prospective multicenter studies are warranted to further validate these findings.

## Data Availability

The original contributions presented in the study are included in the article/supplementary material, further inquiries can be directed to the corresponding author/s.

## References

[1] Dixon JM. Periductal mastitis/duct ectasia. World J Surg. (1989) 13:715–20. doi: 10.1007/BF01658420, 2696225

[2] Bundred NJ. The aetiology of periductal mastitis. Breast. (1993) 2:1–2.

[3] Liang Z Zhang L. Research Progress of plasma cell mastitis. Immun Inflamm Dis. (2025) 13:e70199. doi: 10.1002/iid3.70199, 40289384 PMC12034746

[4] Peters F Kiesslich A Pahnke V. Coincidence of nonpuerperal mastitis and noninflammatory breast cancer. Eur J Obstet Gynecol Reprod Biol. (2002) 105:59–63. doi: 10.1016/S0301-2115(02)00109-4, 12270566

[5] Haitz K Ly A Smith G. Idiopathic granulomatous mastitis. Cutis. (2019) 103:38–42. 30758334

[6] Azizi A Prasath V Canner J Gharib M Sadat Fattahi A Naser Forghani M . Idiopathic granulomatous mastitis: management and predictors of recurrence in 474 patients. Breast J. (2020) 26:1358–62. doi: 10.1111/tbj.13822, 32249491

[7] Wolfrum A Kummel S Theuerkauf I Pelz E Reinisch M. Granulomatous mastitis: a therapeutic and diagnostic challenge. Breast Care (Basel). (2018) 13:413–8. doi: 10.1159/000495146, 30800035 PMC6381909

[8] Freeman CM Xia BT Wilson GC Lewis JD Khan S Lee SJ . Idiopathic granulomatous mastitis: a diagnostic and therapeutic challenge. Am J Surg. (2017) 214:701–6. doi: 10.1016/j.amjsurg.2017.07.002, 28739122

[9] Yilmaz E Lebe B Usal C Balci P. Mammographic and sonographic findings in the diagnosis of idiopathic granulomatous mastitis. Eur Radiol. (2001) 11:2236–40. doi: 10.1007/s003300100965, 11702165

[10] Yuan QQ Xiao SY Farouk O Du YT Sheybani F Tan QT . Management of granulomatous lobular mastitis: an international multidisciplinary consensus (2021 edition). Mil Med Res. (2022) 9:20. doi: 10.1186/s40779-022-00380-5, 35473758 PMC9040252

[11] Martinez-Ramos D Simon-Monterde L Suelves-Piqueres C Queralt-Martin R Granel-Villach L Laguna-Sastre JM . Idiopathic granulomatous mastitis: a systematic review of 3060 patients. Breast J. (2019) 25:1245–50. doi: 10.1111/tbj.13446, 31273861

[12] Lei X Chen K Zhu L Song E Su F Li S. Treatments for idiopathic granulomatous mastitis: systematic review and Meta-analysis. Breastfeed Med. (2017) 12:415–21. doi: 10.1089/bfm.2017.0030, 28731822

[13] Zhou F Liu L Liu L Yu L Wang F Xiang Y . Comparison of conservative versus surgical treatment protocols in treating idiopathic granulomatous mastitis: a Meta-analysis. Breast Care (Basel). (2020) 15:415–20. doi: 10.1159/000503602, 32982653 PMC7490657

[14] Von Elm E Altman DG Egger M Pocock SJ Gotzsche PC Vandenbroucke JP . The strengthening the reporting of observational studies in epidemiology (STROBE) statement: guidelines for reporting observational studies. J Clin Epidemiol. (2008) 61:344–9. doi: 10.1016/j.jclinepi.2007.11.008, 18313558

[15] Tan QT Tay SP Gudi MA Nadkarni NV Lim SH Chuwa EWL. Granulomatous mastitis and factors associated with recurrence: an 11-year single-Centre study of 113 patients in Singapore. World J Surg. (2019) 43:1737–45. doi: 10.1007/s00268-019-05014-x, 31049604

[16] Kamal RM Hamed ST Salem DS. Classification of inflammatory breast disorders and step by step diagnosis. Breast J. (2009) 15:367–80. doi: 10.1111/j.1524-4741.2009.00740.x, 19496780

[17] Going JJ Anderson TJ Wilkinson S Chetty U. Granulomatous lobular mastitis. J Clin Pathol. (1987) 40:535–40. doi: 10.1136/jcp.40.5.535, 3584506 PMC1141020

[18] Pusic AL Klassen AF Scott AM Klok JA Cordeiro PG Cano SJ. Development of a new patient-reported outcome measure for breast surgery: the BREAST-Q. Plast Reconstr Surg. (2009) 124:345–53. doi: 10.1097/PRS.0b013e3181aee807, 19644246

[19] Cano SJ Klassen AF Scott AM Cordeiro PG Pusic AL. The BREAST-Q: further validation in independent clinical samples. Plast Reconstr Surg. (2012) 129:293–302. doi: 10.1097/PRS.0b013e31823aec6b, 22286412

[20] Cohen WA Mundy LR Ballard TN Klassen A Cano SJ Browne J . The BREAST-Q in surgical research: a review of the literature 2009-2015. J Plast Reconstr Aesthet Surg. (2016) 69:149–62. doi: 10.1016/j.bjps.2015.11.013, 26740288 PMC4995882

[21] Cano SJ Klassen AF Scott A Alderman A Pusic AL. Interpreting clinical differences in BREAST-Q scores: minimal important difference. Plast Reconstr Surg. (2014) 134:173e–5e. doi: 10.1097/PRS.0000000000000267, 25028843

[22] Ferreira-Valente MA Pais-Ribeiro JL Jensen MP. Validity of four pain intensity rating scales. Pain. (2011) 152:2399–404. doi: 10.1016/j.pain.2011.07.005, 21856077

[23] Hjermstad MJ Fayers PM Haugen DF Caraceni A Hanks GW Loge JH . Studies comparing numerical rating scales, verbal rating scales, and visual analogue scales for assessment of pain intensity in adults: a systematic literature review. J Pain Symptom Manag. (2011) 41:1073–93. doi: 10.1016/j.jpainsymman.2010.08.016, 21621130

[24] Farrar JT Young JP Jr LaMoreaux L Werth JL Poole MR. Clinical importance of changes in chronic pain intensity measured on an 11-point numerical pain rating scale. Pain. (2001) 94:149–58. doi: 10.1016/S0304-3959(01)00349-9, 11690728

[25] Melzack R. The McGill pain questionnaire: major properties and scoring methods. Pain. (1975) 1:277–99. doi: 10.1016/0304-3959(75)90044-5, 1235985

[26] Beaton DE Wright JG Katz JN Upper Extremity Collaborative Group. Development of the QuickDASH: comparison of three item-reduction approaches. J Bone Joint Surg Am. (2005) 87:1038–46. doi: 10.2106/JBJS.D.02060, 15866967

[27] LeBlanc M Stineman M DeMichele A Stricker C Mao JJ. Validation of QuickDASH outcome measure in breast cancer survivors for upper extremity disability. Arch Phys Med Rehabil. (2014) 95:493–8. doi: 10.1016/j.apmr.2013.09.016, 24095658 PMC4216590

[28] Franchignoni F Vercelli S Giordano A Sartorio F Bravini E Ferriero G. Minimal clinically important difference of the disabilities of the arm, shoulder and hand outcome measure (DASH) and its shortened version (QuickDASH). J Orthop Sports Phys Ther. (2014) 44:30–9. doi: 10.2519/jospt.2014.4893, 24175606

[29] Finley M Jelinek JA Misamore G. Three-dimensional analysis versus goniometric measurement of total active elevation in normal subjects. J Shoulder Elb Surg. (2015) 24:1391–6. doi: 10.1016/j.jse.2015.02.005, 25825139

[30] Ware JE Sherbourne CD. The MOS 36-item short-form health survey (SF-36). I. Conceptual framework and item selection. Med Care. (1992) 30:473–83. doi: 10.1097/00005650-199206000-00002, 1593914

[31] Treanor C Donnelly M. A methodological review of the short form health survey 36 (SF-36) and its derivatives among breast cancer survivors. Qual Life Res. (2015) 24:339–62. doi: 10.1007/s11136-014-0785-6, 25139502

[32] Laucis NC Hays RD Bhattacharyya T. Scoring the SF-36 in Orthopaedics: a brief guide. J Bone Joint Surg Am. (2015) 97:1628–34. doi: 10.2106/JBJS.O.00030, 26446970 PMC5029523

[33] Ogura K Yakoub MA Christ AB Fujiwara T Nikolic Z Boland PJ . What are the minimum clinically important differences in SF-36 scores in patients with Orthopaedic oncologic conditions? Clin Orthop Relat Res. (2020) 478:2148–58. doi: 10.1097/CORR.0000000000001341, 32568896 PMC7431256

[34] Fitzmaurice GM Lard NM Ware JH Laird NM Ware JH Lard NM . Applied Longitudinal Analysis. New York: John Wiley & Sons (2004).

[35] Singer JD Willet JB. Applied Longitudinal Data Analysis: Modeling Change and Event Occurrence. Part I. Oxford: Oxford University Press (2003).

[36] Little RJ. Missing Data Analysis. Annu Rev Clin Psychol. (2024) 20:149–73. doi: 10.1146/annurev-clinpsy-080822-051727, 38346291

[37] Cohen J. Statistical Power Analysis for the Behavioral Sciences. 2nd ed. Cambridge, MA: Academic Press (1988).

[38] Kreidler SM Muller KE Grunwald GK Ringham BM Coker-Dukowitz ZT Sakhadeo UR . GLIMMPSE: online power computation for linear models with and without a baseline covariate. J Stat Softw. (2013) 54:10. doi: 10.18637/jss.v054.i10, 24403868 PMC3882200

[39] Molenberghs G Kenward MG *Missing Data in Clinical Studies: Missing Data in Clinical Studies* (2007).

[40] Buuren SV. Flexible Imputation of Missing Data. Boca Raton, FL: CRC Press (2012).

[41] Baslaim MM Khayat HA Al-Amoudi SA. Idiopathic granulomatous mastitis: a heterogeneous disease with variable clinical presentation. World J Surg. (2007) 31:1677–81. doi: 10.1007/s00268-007-9116-1, 17541683

[42] Deng Y Xiong Y Ning P Wang X Han XR Tu GF . A case management model for patients with granulomatous mastitis: a prospective study. BMC Womens Health. (2022) 22:143. doi: 10.1186/s12905-022-01726-w, 35501850 PMC9063211

[43] Mundy LR Homa K Klassen AF Pusic AL Kerrigan CL. Breast Cancer and reconstruction: normative data for interpreting the BREAST-Q. Plast Reconstr Surg. (2017) 139:1046e–55e. doi: 10.1097/PRS.0000000000003241, 28445351 PMC5713639

[44] Molle M Uderzo S D’Addato C Gesuete FP Nicoletti GF Ferraro GA. Is quality of life after breast reduction influenced by different surgery’s technique? A prospective study. Aesthet Surg J. (2024) 48:4973–8. doi: 10.1007/s00266-024-04050-w, 38769147

[45] Mazzarella V Molle M Auriemma E Cimmino M Faenza M. Lateral based dermal flap in breast contouring in reduction mammaplasty. Int J Surg Case Rep. (2025) 127:110876. doi: 10.1016/j.ijscr.2025.110876, 39827658 PMC11786090

